# The combination of decoy receptor 3 and soluble triggering receptor expressed on myeloid cells-1 for the diagnosis of nosocomial bacterial meningitis

**DOI:** 10.1186/s12941-015-0078-0

**Published:** 2015-03-23

**Authors:** Yong-Juan Liu, Li-Hua Shao, Jian Zhang, Shan-Ji Fu, Gang Wang, Feng-Zhe Chen, Feng Zheng, Rui-Ping Ma, Hai-Hong Liu, Xiao-Meng Dong, Li-Xian Ma

**Affiliations:** Department of Infectious Diseases, Qilu Hospital of Shandong University, Wenhua Xi Road 107, 250012 Jinan, Shandong China; Department of Laboratory Sciences, School of Public Health of Shandong University, Wenhua Xi Road 44, Jinan, 250012 China; Department of Clinical Laboratory, Qilu Hospital of Shandong University, Wenhua Xi Road 107, Jinan, 250012 China; Department of Digestive System Diseases, Shandong Provincial Qianfoshan Hospital of Shandong University, Jingshi Road 16766, Jinan, 250014 China; Shandong Center for Disease Control and Prevention, Jingshi Road 16992, Jinan, 250014 China

**Keywords:** Bacterial meningitis, Diagnosis, DcR3, sTREM-1, Nosocomial infection

## Abstract

**Background:**

Early diagnosis and appropriate antibiotic treatment can significantly reduce mortality of nosocomial bacterial meningitis. However, it is a challenge for clinicians to make an accurate and rapid diagnosis of bacterial meningitis. This study aimed at determining whether combined biomarkers can provide a useful tool for the diagnosis of bacterial meningitis.

**Methods:**

A retrospective study was carried out. Cerebrospinal fluid (CSF) levels of decoy receptor 3 (DcR3) and soluble triggering receptor expressed on myeloid cells-1 (sTREM-1) were detected by enzyme-linked immunosorbent assay (ELISA).

**Results:**

The patients with bacterial meningitis had significantly elevated levels of the above mentioned biomarkers. The two biomarkers were all risk factors with bacterial meningitis. The biomarkers were constructed into a “bioscore”. The discriminative performance of the bioscore was better than that of each biomarker, with an area under the receiver operating characteristic (ROC) curve (AUC) of 0.842 (95% confidence intervals (CI) 0.770–0.914; *p*< 0.001).

**Conclusions:**

Combined measurement of CSF DcR3 and sTREM-1 concentrations improved the prediction of nosocomial bacterial meningitis. The combined strategy is of interest and the validation of that improvement needs further studies.

## Introduction

Nosocomial bacterial meningitis is a significant problem among hospitalized patients, which threatens patients’ lives, extends their stay in hospital and increases the medical costs, sometimes even results in doctor-patient conflicts. Despite emergence of antibiotics and improvement of clinical techniques, bacterial meningitis continues to be a significant cause of morbidity and mortality [1,2]. Early effective antibiotic therapy is associated with better outcomes [3,4] and the choice of appropriate antibiotics is greatly influenced by the final identification of the specific pathogen.

However, the diagnosis of this disease remains a challenge. Firstly, the clinical signs of fever, headache, neck stiffness and mental status alteration are not specific [1,2]. Secondly, laboratory tests are also not specific enough. Cerebrospinal fluid (CSF) culture is specific but lacks sensitivity, especially with previous use of antibiotics [2]. In addition, the unnecessary use of antibiotics increases the chance of being infected by multidrug-resistant bacteria [4]. Based on the above reasons, the development of new indicators for the rapid diagnosis of bacterial meningitis is desirable.

Decoy receptor 3 (DcR3), a soluble decoy receptor in the TNF receptor (TNFR) family, is capable of inhibiting apoptosis by neutralizing the activities of the following three members of the TNF superfamily: Fas ligand (FasL) [5], LIGHT [receptor homologous to lymphotoxins exhibits inducible expression, competes with herpes simplex virus (HSV) glycoprotein D for HVEM and is expressed by T lymphocytes] [6] and TNF-like molecule 1A (TL1A) [7]. In addition, DcR3 can be a new potential biomarker for inflammatory disease, autoimmune diseases and cancer [8-12]. Our previous study has proved that levels of DcR3 are significantly elevated in patients with bacterial meningitis and it may act as a useful biomarker of bacterial meningitis [13].

The triggering receptor expressed on myeloid cells-1 (TREM-1) is a recently described receptor on the surfaces of monocytes and neutrophils [14]. Many previously published studies have indicated that the soluble form of TREM-1, called sTREM-1, is a useful indicator for bacterial infection [15-18]. Indeed, several studies have suggested that sTREM-1 is valuable for the diagnosis of bacterial meningitis [16,17] before a positive result of CSF culture. These data show the individual predictive value of these two biomarkers. Combination of several biomarkers can improve diagnostic value in various diseases or conditions: such as sepsis [19], liver fibrosis [20], community-acquired pneumonia [21] and severe malaria [22]. However, the strategy of combined DcR3 and sTREM-1 has not yet been validated for bacterial meningitis.

The aim of this study was to evaluate the combined diagnostic value of DcR3 and sTREM-1.

## Materials and methods

### Patients and data collection

Diagnosis of bacterial meningitis was based on a positive result of CSF culture. Nosocomial meningitis was defined as negative bacterial infection when the patient was admitted to the hospital, clinical evidence of an infection was found after 48 hours on admission or within one month after discharge from the hospital where the patient had received an invasive neurosurgical procedure. Otherwise, the patient was considered to have community-acquired meningitis [23,24]. External ventricular drain-related meningitis was diagnosed with bacterial infection being found within 7 days of external ventricular drain removal [25].

One hundred and twenty-three patients in this study were enrolled in Qilu Hospital of Shandong University in China within November 2012 and October 2013. All patients in this study were categorized into two groups. Inclusion and exclusion criteria are described more extensively elsewhere. The mean age (mean ± SD) of 80 patients with bacterial meningitis was 43.75 ± 16.46 years. The area under the receiver operating characteristic (ROC) curve (AUC) of DcR3 was 0.831 [13]. The biomarkers associated with this study were CSF DcR3 and sTREM-1.

This study was approved by Institutional Research Ethics Committee of Qilu Hospital of Shandong University (No. KYLL-2012-096). Written informed consents were taken from patients or clients before registration.

### Detection of sTREM-1 and DcR3

The CSF samples were centrifuged and supernatants were frozen at −80°C until assay. Levels of sTREM-1 and DcR3 in CSF were determined in duplicate by ELISA according to the kits instructions (Cusabio, Wuhan, China). The detection limits were 7.8 pg/mL for sTREM-1 and 0.039 ng/mL for DcR3.

### Statistical analysis

Descriptive results of sTREM-1 and bioscore were expressed as median (25th and 75th percentiles) and Mann–Whitney *U*-test was used to analyze the continuous data of sTREM-1 and bioscore. ROC curve and AUC were applied to evaluate the discriminatory power of each marker and combined CSF markers. The Youden index (*J* = max (sensitivity + specificity − 1)) was used to define the cut-off value. Risk factors related to bacterial meningitis were explored by multivariate stepwise logistic regression analysis. Hosmer-Lemeshow goodness-of-fit test was used for the calibration of logistic regression model. All analyses were performed with SPSS, version 20.0 and a two-sided *p* < 0.05 was considered to be statistically significant.

## Results

### Demographic characteristics of patients

Among the 123 patients recruited in this study, 80 patients were diagnosed with bacterial meningitis [13]. In patients with bacterial meningitis, 6 patients had external ventricular drain-related meningitis. The levels of CSF DcR3 were significantly different between the groups of bacterial meningitis and non-bacterial meningitis. 44 patients received antibiotic >24 h before CSF sampling, among them 5 patients with non-bacterial meningitis and 39 patients with bacterial meningitis [13]. Concentrations of sTREM-1 in the patients with bacterial meningitis were significantly higher than those with non-bacterial meningitis (19.017 (0 –60.256) pg/mL *vs.* 0 (0–0) pg/mL, *p* < 0.001).

As shown in Table 1, predisposing conditions were present in all patients, such as neurosurgery, pneumonia, CSF leak, *etc.* All the 80 patients with bacterial meningitis underwent neurosurgical operation (Tables 1 and 2). The most frequently isolated microorganism was coagulase-negative Staphylococci (Table 3). More than half of the patients (n=44) had intracranial tumor surgery. The conditions necessitating surgical interventions were shown in Table 2.

**Table 1:** **Characteristics of patients with bacterial meningitis**

**Characteristics**	**Bacterial meningitis (n=80)**
Predisposing Factors	80
Pneumonia	7 (8.75)
Diabetes	6 (7.50)
Neurosurgery	80(100)
Recent Head Injury*	6 (7.50)
CSF Leak	4 (5.00)
Neurosurgical Devices^▲^	44 (55.00)
Symptoms and Signs on Presentation	
Seizures	6 (7.50)
Headache	57 (71.25)
Neck Stiffness	28 (35.00)
Body Temperature≥ 38°C	63 (78.75)
Triad of Fever, Neck Stiffness, and Change in Mental Status	10 (12.50)
Positive Blood Culture	1/3 (33.33)^☼^

All categorical data are expressed as number (%) unless indicated otherwise. *Recent denotes within one month of the onset of meningitis. CSF: cerebrospinal fluid. ^▲^Neurosurgical devices include ventriculostomy, ventriculoperitoneal shunt, lumbar puncture catheter, spinal cavity shunt and external ventricular drainage catheter. ^☼^Data are number/number evaluated (%).

**Table 2:** **Neurosurgical interventions**

**Type of surgery**	**Positive culture (n=80)**
Intracranial Tumor Operation	44
Evacuation of Intracranial Haematoma	6
Repair of Cranial Defect	6
Ventriculoperitoneal Shunt	4
Repair of CSF Rhinorrhea	1
Suboccipital Decompression	4
Ventriculostomy	3
Decompression of Trigeminal Neuralgia	3
Ventricular External Drainage	3
Aneurysm Plus Ligation	1
Aneurysm Embolization	3
Arachnoid Cyst Excision	1
Posterior Fossa Decompression and Spinal Cavity Shunt	1

**Table 3:** **Results of isolated microorganism(s)**

**Microorganism(s) Isolated**	**Positive Culture (n)**
Coagulase-Negative Staphylococci	62
Staphylococcus aureus	4
Pediococcus pentosaceus	1
Enterobacter cloacae	3
Pseudomonas aeruginosa	2
Klebsiella pneumoniae	2
Escherichia coli	2
Bacillus typhi suis	1
Acinetobacter baumannii	1
Coagulase-Negative Staphylococci and Acinetobacter baumannii	1
Pseudomonas oryzihabitans	1

### Evaluation of sTREM-1 and DcR3 in discriminating bacterial meningitis from non-bacterial meningitis

**Figure 1:**
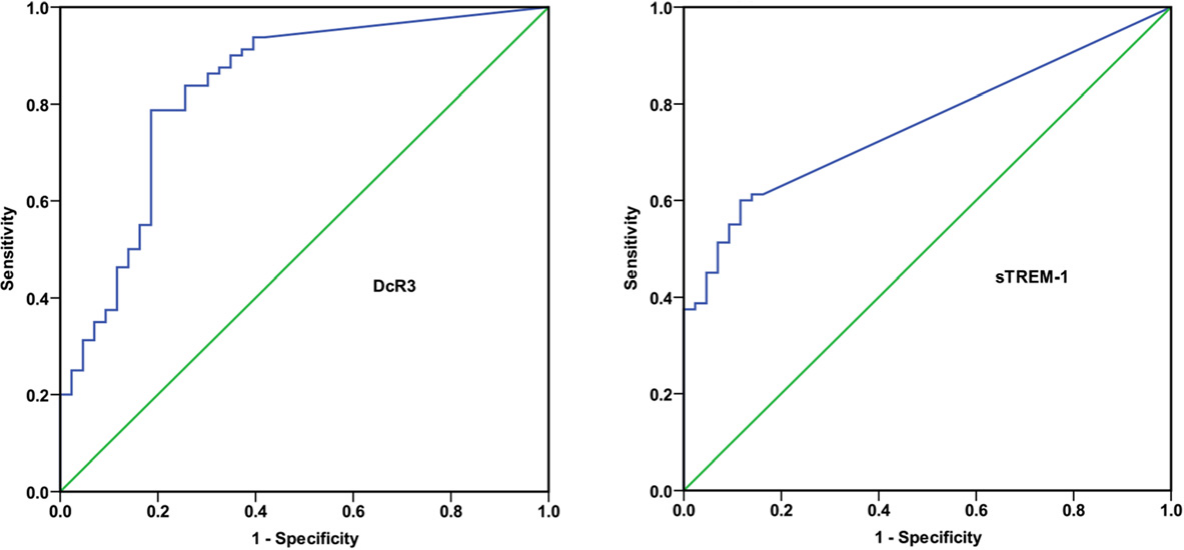
**Receiver operating characteristic (ROC) curves of DcR3 and sTREM-1 for the diagnosis of bacterial meningitis.** Areas under the ROC curve (AUC) are presented with 95% confidence intervals (CI). The AUC of DcR3 has been published previously [13]. In order to compare the AUC of sTREM-1 with that of DcR3, the figure of DcR3 is presented here. DcR3: 0.831(95% CI 0.752–0.911); sTREM-1: 0.756 (95% CI 0.673-0.839).

ROC curve and AUC were performed to determine the discriminative accuracy of CSF sTREM-1 and DcR3. Combined with the published data, CSF DcR3 yielded a higher discriminative value with an AUC of 0.831 [13]. The AUC of sTREM-1 for predicting bacterial meningitis was 0.756 (95% confidence intervals (CI) 0.673-0.839; *p* < 0.001, Figure 1). A cut-off value of 11.515 pg/mL for sTREM-1 had a sensitivity of 60.00% (95 CI 48.42% – 70.61%), a specificity of 88.37% (95% CI 74.12% – 95.64%), a positive likelihood ratio (PLR) of 5.16 (95% CI 2.22-11.99), a negative likelihood ratio (NLR) of 0.45 (95% CI 0.34-0.60), a positive predictive value (PPV) of 90.57% (95% CI 78.58%-96.47%) and a negative predictive value (NPV) of 54.29% (95% CI 42.01%-66.09%).

### Comparison of DcR3 and sTREM-1 with other markers in predicting bacterial meningitis

As shown in Table 4, CSF leucocyte count yielded the best discriminative value, with an AUC of 0.928, a sensitivity of 88.75% and a specificity of 90.70%. Multivariate stepwise logistic regression was applied to evaluate the independent predictors for bacterial meningitis. From univariate analysis, the following 6 variables: CSF leucocyte count, glucose, protein, lactate, DcR3 and sTREM-1 were associated with bacterial meningitis (Table 5). All the six markers were taken into a multivariate stepwise logistic regression model. The result showed that only sTREM-1 and DcR3 were the independently risk factors with bacterial meningitis (odds ratio (OR) = 3.325, 95% CI = 1.185–9.334, *p* = 0.023 for DcR3; OR = 1.059, 95% CI = 1.015–1.106, *p* = 0.008 for sTREM-1; Table 6).

**Table 4:** **Diagnostic performance of DcR3, sTREM-1 and other CSF markers for the diagnosis of bacterial meningitis**

**Variable**	**Area under ROC Curve**	**Sensitivity (%)**	**Speificity (%)**
DcR3	0.831	78.75	81.40
sTREM-1	0.756	60.00	88.37
Leucocyte Count	0.928	88.75	90.70
Glucose	0.696	65.00	67.44
Protein	0.664	43.75	86.05
Lactate	0.717	62.50	74.42

The performance of DcR3 and other CSF markers has been published previously [13]. In order to compare the diagnostic performance of the two biomarker with that of other CSF markers, the data of DcR3 and CSF markers are presented here. ROC: receiver operating characteristic.

**Table 5:** **Univariate analysis for diagnosing bacterial meningitis**

**Variable**	**Coefficient**	**SE**	**Wald**	**df**	***p*** **value**	**OR (95% CI)**
DcR3	2.073	0.528	15.401	1	<0.001	7.949 (2.823-22.385)
sTREM-1	0.071	0.020	11.871	1	0.001	1.073 (1.031-1.117)
Leucocyte Count	0.003	0.001	13.690	1	<0.001	1.003 (1.001-1.004)
Glucose	−0.428	0.151	8.013	1	0.005	0.652 (0.485-0.877)
Protein,	0.673	0.264	6.528	1	0.011	1.961 (1.170-3.286)
Lactate	0.375	0.117	10.355	1	0.001	1.455 (1.158-1.829)

SE= standard error; df= degrees of freedom; OR= odds ratio.

**Table 6:** **Multiple logistic regression analysis of risk factors used for diagnosing bacterial meningitis**

							**Hosmer-Lemeshow test**		
**Variable**	**Coefficient**	**SE**	**Wald**	**df**	***p*** **value**	**OR (95% CI)**	**Chi Square**	**df**	***p*** **value**
Model 1^●^									
DcR3	1.202	0.527	5.206	1	0.023	3.325 (1.185-9.334)	4.060	8	0.852
sTREM-1	0.058	0.022	6.943	1	0.008	1.059 (1.015-1.106)			
Model 2^☼^									
Biscore	1.947	0.343	32.180	1	<0.001	7.007 (3.576-13.730)	0.079	1	0.779

SE= standard error; df= degrees of freedom; OR= odds ratio.

Model 1: each biomarker is entered into the model.

Model 2: biomarkers are combined into the bioscore that is then entered into the model.

^●^Pseudo R^2^ (Cox and Snell) 0.326.

^☼^Pseudo R^2^ (Cox and Snell) 0.337.

### Combination of DcR3 and sTREM-1 to predict bacterial meningitis

In order to determine whether combined detection of the markers mentioned above could improve diagnostic accuracy, the biomarkers were combined into a “bioscore”. For each biomarker, individual data were scored as 1 or 0. If the data were higher than the critical value, the scores were recorded as 1; and if the data were below the cut-off point, the scores were recorded as 0. The scores constituted the cumulative bioscore, which ranged between 0 and 2.

**Figure 2:**
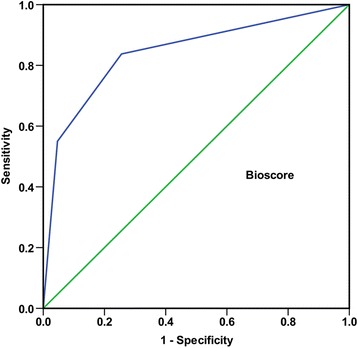
**Receiver operating characteristic (ROC) curve of bioscore for the diagnosis of bacterial meningitis.** The data is presented with 95% confidence interval (CI). Bioscore: 0.842 (95% CI 0.770–0.914).

The biomarker score was significantly elevated for patients with bacterial meningitis (2 (1–2) *vs.* 0 (0 –1), *p* < 0.001). ROC curve was constructed as above for the bioscore. The AUC for bioscore was 0.842 (95% CI 0.770–0.914; *p*< 0.001; Figure 2). When the bioscore was entered into the multivariate stepwise logistic regression model, the bioscore was also proved to be a significant factor for bacterial meningitis (OR, 7.007, 95% CI 3.576–13.730; *p*< 0.001, Table 6).

**Figure 3:**
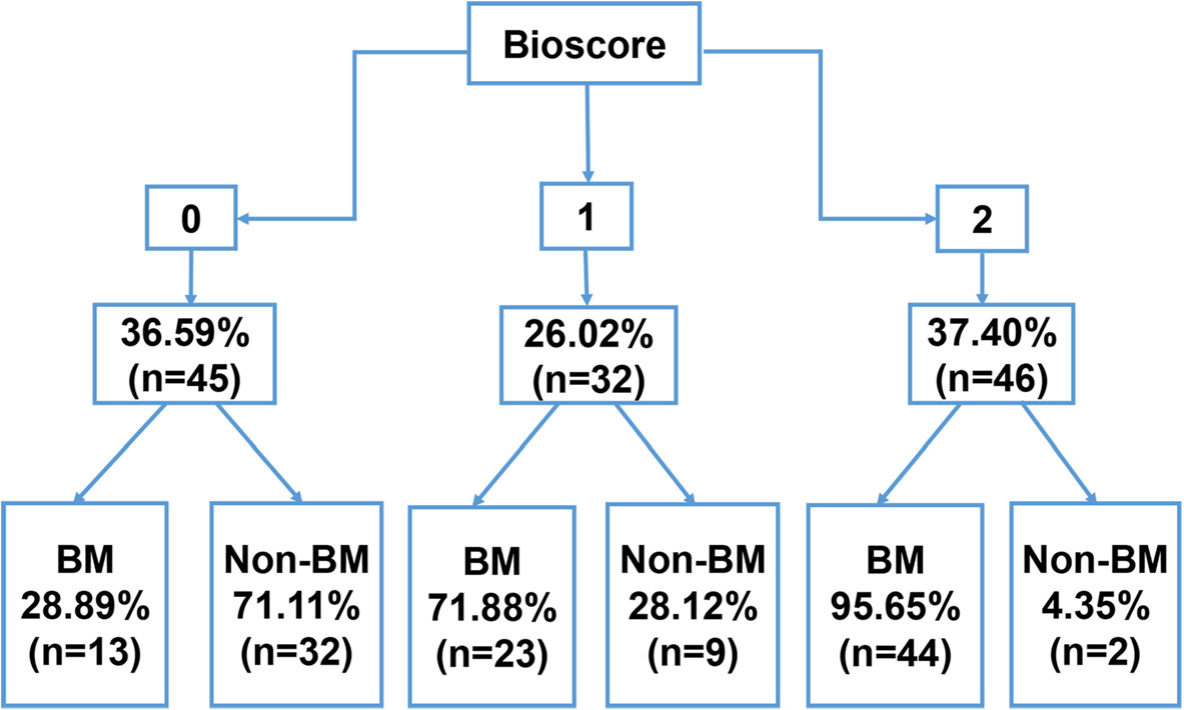
**Group of patients according to the bioscore. BM: bacterial meningitis; Non-BM: non-bacterial meningitis.** The types of organisms according to the bioscore are as follows: Bioscore 0, coagulase-negative Staphylococci (n=10), Staphylococcus aureus (n=2) and Pseudomonas aeruginosa (n=1); Bioscore 1, coagulase-negative Staphylococci (n=18), Staphylococcus aureus (n=1), Pediococcus pentosaceus (n=1), Klebsiella pneumoniae (n=1), Bacillus typhi suis (n=1), a mix infection of coagulase-negative Staphylococci and Acinetobacter baumannii (n=1); Bioscore 2, coagulase-negative Staphylococci (n=34), Staphylococcus aureus (n=1), Enterobacter cloacae (n=3), Pseudomonas aeruginosa (n=1), Klebsiella pneumoniae (n=1), Escherichia coli (n=2), Acinetobacter baumannii (n=1) and Pseudomonas oryzihabitans (n=1).

The possibility of being infected by bacteria grew with the increasing bioscore. The rate of bacterial meningitis ranged from 28.89% for a bioscore of 0 to 95.65% for a bioscore of 2. According to the bioscore, the patients were divided into three groups. 67 of 78 patients (85.90%) with a bioscore of 1 or 2 were diagnosed with bacterial meningitis (Figure 3).

## Discussion

Combination of several markers has been found to improve predictive value in various disorders [19-22]. In this study, we combined the markers of DcR3 and sTREM-1 into a simple score, named as “bioscore”, which was proved to be a useful predictor for the diagnosis of bacterial meningitis. Furthermore, compared with the individual marker of DcR3 or sTREM-1, the bioscore was found to be a better predictor of bacterial meningitis.

Except its neutralizing effects on FasL, LIGHT, and TL1A, DcR3 also acts as a pleiotropic effector which can regulate cellular function via “non-decoy” activities [9]. In addition, DcR3 may have pro-inflammatory functions [26,27]. In ARDS patients, higher plasma DcR3 levels are associated with multiple-organ dysfunction, longer duration of ICU stay and ventilator dependence. Serum DcR3 levels in non-survivors are higher than those in survivors regardless of the APACHE II scores. Therefore, DcR3 appears to possess the potential to serve as both a diagnostic and a prognostic marker of ARDS [28]. Moreover, levels of DcR3 are elevated in patients with sepsis and it may act as a clinically important biomarker of sepsis. DcR3 might be considered as a double-edged sword in sepsis and have the potential to be a novel target for the treatment of sepsis [10,12]. Our previous study has showed that detection of CSF DcR3 is useful for the diagnosis of bacterial meningitis. A cut-off point of 0.201 ng/mL is established, which has a sensitivity of 78.75% and a specificity of 81.40% [13]. Thus, similar to sepsis, DcR3 might turn out to be a novel target of bacterial meningitis. Further studies are needed to determine the confirmatory role of abnormally high level of DcR3 in CSF of patients with bacterial meningitis.

As previously published studies [16,17], sTREM-1 was also found significantly increased in patients with bacterial meningitis in present study. However, there are some discrepancies between our data and previously published data. CSF sTREM-1 has a diagnostic AUC of 0.82, a sensitivity of 73% and a specificity of 77% [16]. Sensitivity of the study by Bishara J and coworkers is 77.8%, while the specificity is as high as 100% [17]. However, the sensitivity of sTREM-1 in our study was poorer, only with a sensitivity of 60.00%.

Several potential explanations for these discrepancies were as follows:The first explanation may be the difference of enrolled patients. The population enrolled in this study only included patients with nosocomial bacterial meningitis. The study by Determann RM *et al.* has 109 patients, among them 92 patients being diagnosed with bacterial meningitis, 8 with viral meningitis and 9 healthy donors. Only patients with community-acquired bacterial meningitis are recruited [16]. Another study by Bishara J and coworkers has two groups and only has 21patients, including 9 patients with a positive culture and 12 patients with a negative culture, which is much less than that of this study. Moreover, the study doesn’t have the limit of WBC count [17]. Another important explanation might be technical. Except for the ELISA kits from different manufacturers, the sensitivity was different, which might lead to a lower cut- off value of sTREM-1 in present study.

In addition to the individual diagnostic accuracy of each biomarker, the combination of DcR3 and sTREM-1 into a bioscore appeared to be an efficient and practical way to discriminate patients with bacterial meningitis from patients with non-bacterial meningitis. The OR of biscore was 7.007. The same as other disorders or diseases, such as sepsis and severe malaria [19,22], the higher the score was recorded, the greater the possibility of being infected by bacteria was determined. Bioscore 2 could yield 95.65% of patients with bacterial meningitis. With at least one of the two biomarkers higher than the critical point (bioscore ≥1), about 86% of patients were found to be infected by bacteria. Nonetheless, with a bioscore of 0, 28.89% of patients could not be excluded. In addition, because serum of patients in this study were not collected, predictive value of combined bioscore in blood for bacterial meningitis was not determined. Further studies are required to validate the potential value of combined bioscore in serum for the diagnosis of bacterial meningitis.

Currently, CSF culture is still the golden standard for the diagnosis of bacterial meningitis. Early diagnosis of bacterial meningitis is still a challenging problem for clinicians. In this study, although the negative predictive value of bioscore 0 was not satisfactory, a strong positive predictive value was found if the bioscore was above 1. It seemed likely that the combined bioscore could strengthen the clinicians’ decisions besides clinical work-up.

Our study had two major limitations. (1) This retrospective study included unsatisfactorily sample size and firm conclusions were not concluded. The selection of non-consecutive samples might have resulted in a selection bias. Predictive value of combined DcR3 and sTREM-1 needs validation in larger-scale prospective studies. (2) Only bacterial culture was used to determine the presence of bacterial meningitis. 11%–30% of patients with bacterial meningitis are with negative results of bacterial culture [1]. Therefore, some patients with non-bacterial meningitis might be misclassified and limit the application of our findings.

## Conclusions

In conclusion, this retrospective study demonstrated that combination of DcR3 and sTREM-1 in CSF could yielded a better diagnostic value for nosocomial bacterial meningitis than that of each biomarker. Whether the bioscore be applied routinely in clinic needs further studies.
